# Restoration of caveolin-1 expression suppresses growth and metastasis of head and neck squamous cell carcinoma

**DOI:** 10.1038/sj.bjc.6604735

**Published:** 2008-10-28

**Authors:** H Zhang, L Su, S Müller, M Tighiouart, Z Xu, X Zhang, H J C Shin, J Hunt, S-Y Sun, D M Shin, Z(G) Chen

**Affiliations:** 1 Department of Hematology and Medical Oncology, Winship Cancer Institute, Emory University School of Medicine, Atlanta, GA, USA; 2 Department of Pathology, Emory University School of Medicine, Atlanta, GA, USA; 3 Department of Biostatistics and Bioinformatics, Emory University Rollins School of Public Health, Atlanta, GA, USA; 4 Quest Diagnostics, Atlanta, GA, USA; 5 Department of Anatomic Pathology, The Cleveland Clinic, Cleveland, OH, USA

**Keywords:** squamous cell carcinoma of the head and neck, caveolin-1, integrin *β*1, Src

## Abstract

Caveolin-1 (Cav-1) plays an important role in modulating cellular signalling, but its role in metastasis is not well defined. A significant reduction in Cav-1 levels was detected in lymph node metastases as compared with primary tumour of head and neck squamous cell carcinoma (HNSCC) specimens (*P*<0.0001), confirming the downregulation of Cav-1 observed in a highly metastatic M4 cell lines derived from our orthotopic xenograft model. To investigate the function of Cav-1 in metastasis of HNSCC, we compared stable clones of M4 cells carrying human *cav-1* cDNA (CavS) with cells expressing an empty vector (EV) *in vitro* and in the orthotopic xenograft model. Overexpression of Cav-1 suppressed growth of the CavS tumours compared with the EV tumours. The incidence of lung metastases was significantly lower in animals carrying CavS tumours than those with EV tumours (*P*=0.03). *In vitro*, CavS cells displayed reduced cell growth, invasion, and increased anoikis compared with EV cells. In CavS cells, Cav-1 formed complex with integrin *β*1 and Src. Further application of integrin *β*1 neutralising antibody or Src inhibitor PP2 to EV cells illustrated similar phenotypes as CavS cells, suggesting that Cav-1 may play an inhibitory role in tumorigenesis and lung metastasis through regulating integrin *β*1- and Src-mediated cell–cell and cell–matrix interactions.

Caveolin-1 (Cav-1) is a 21–24 kDa integral membrane protein and a principal structural component of caveolae. A scaffolding amino-acid sequence identified in Cav-1 allows this protein to interact with signalling molecules such as epidermal growth factor receptor, G-proteins, c-Src-like kinases, HA-Ras, protein kinase C, endothelial nitric-oxide synthase, and integrin (Razani *et al*, 2001; Liu *et al*, 2002). Substantial evidence of increased expression of Cav-1 associated with tumour progression and metastasis has been established mostly in prostate cancer (Williams *et al*, 2005). In contrast, it has been suggested that Cav-1 acts as a putative tumour and metastasis suppressor of breast cancer (Sloan *et al*, 2004; Williams *et al*, 2004; Williams and Lisanti, 2005), indicating that the role of Cav-1 in metastasis is cell-type specific and remains to be defined. Thus, comprehensive studies of the loss of Cav-1 expression and the role of Cav-1 in metastasis development are lacking.

Squamous cell carcinoma (SCC) represents more than 90% of head and neck (HN) malignant tumours. Development of metastasis, particularly distant metastasis, is a major obstacle to the successful treatment of these patients. However, the genetic mechanisms contributing to behaviour of metastatic cells in human HNSCC are not well understood. A unique metastasis mouse model established in our laboratory represents a late stage of metastasis development in human HNSCC (Zhang *et al*, 2002). Cell lines derived through the fourth round of *in vivo* selection generated a significantly greater incidence of lymph node and lung metastases than their parental cells (Zhang *et al*, 2006). cDNA microarray analysis of both non-metastatic and highly metastatic HNSCC cells revealed that significant downregulation of Cav-1 and -2, loss of E-cadherin and p53 and a marked increase in integrin *β*1 protein levels were associated with highly metastatic behaviour (Zhang *et al*, 2006). In addition, differentially altered proteins identified from the cDNA microarray analysis, including E-cadherin, EGFR, S100A2, protease-activated receptor-1, and CCR7, were confirmed in human HNSCC tissue samples (Wang *et al*, 2004; Zhang *et al*, 2004a, 2007; Muller *et al*, 2008). These observations suggest that this model represents at least one population of metastatic HNSCC cells, thus providing an excellent means to examine the role of Cav-1 in tumour progression and metastasis potential in HNSCC.

By using a HNSCC metastasis animal model, this study presents that the downregulation of Cav-1 observed in cell lines derived from the *in vivo* selection is consistent with that in human tissue specimens. Expression of Cav-1 was inversely associated with metastasis of HNSCC, but positively associated with tumour differentiation. Further *in vitro* studies revealed that Cav-1 protein can directly interact with integrin *β*1 and Src, thus disrupting integrin *β*1/Src-mediated tumour cell growth, invasion, and survival during metastasis. Most importantly, the study has revealed the novel findings of the differential effect of Cav-1 on lymph node and lung metastasis in the animal model. Overexpression of Cav-1 sensitises metastatic cells to anoikis, ultimately leading to reduced lung metastasis.

## Materials and methods

### Tumour specimens and patient information

Consent for tissue acquisition was approved by Institutional Review Board of University of Pittsburgh. A total of 114 specimens were randomly collected from surgical specimens obtained between 1983 and 1993. Requirements for inclusion in the study included no previous radiation or chemotherapy treatment before the resection and that paraffin blocks were available for study. The collected formalin-fixed paraffin-embedded tissue blocks consisted of three groups: primary tumours with positive lymph nodes (Tu-1), their paired lymph node metastases (LNM-1), and primary tumours with negative lymph nodes (Tu-2). The number of samples and distribution were *n*=34 (Tu-1), *n*=40 (LNM-1), *n*=40 (Tu-2). Tumour grade was categorised as poorly differentiated (PD), moderately differentiated (MD), and well differentiated (WD) by two pathologists (SM and HJCS) independently. Eleven normal oral epithelial tissue samples from non-cancer patients were used as a control. The clinical information was available for some of the samples (*n*=57) and was obtained from the surgical pathology files at the University of Pittsburgh, following the regulations of the Health Insurance Portability and Accountability Act. Of these patients, 17 had stage T1–T2 and 23 had T3–T4 disease. Primary tumour sites included oral cavity (floor of mouth, tongue, retromolar trigone, and alveolar ridge, *n*=16), pharynx (soft palate, base of tongue, and posterior wall of pharynx, *n*=6), and larynx (aryepiglottic fold, glottis, epiglottis, subglottic, and transglottic, *n*=18). There were equal numbers of males and females with an average age of 61.2 years and median months of follow-up 72 (ranging from 8 to 172 months). At the time of data collection, 9 patients showed no evidence of disease, 12 had died of complications, and 16 had died of disease.

### Cell lines

The HNSCC cell line 686LN was established from a lymph node metastasis of a primary base of tongue tumour (Sacks, 1996). 686LN has a low capability for metastasis to the lymph node and none to the lung in nude mice as described previously (Zhang *et al*, 2002, 2006). Derived through *in vivo* selection in nude mice from the parental 686LN cells, 686LN-M3s (M3s), and 686LN-M4 s (M4 s) cells, including the clones M3a, M3b, M4c, M4d, and M4e, are highly metastatic cell lines capable of generating high incidences of lymph node and lung metastases. The cell lines were maintained as monolayer cultures in Dulbecco's modified Eagle's medium (DMEM)/F12 medium (1 : 1) supplemented with 10% fetal bovine serum (FBS) at 37°C in a humidified atmosphere with 5% CO_2_.

### Chemicals and antibodies

Antibodies and their sources were as follows: polyclonal anti-Cav-1 (sc-894), -MMP-2, and -p27 were purchased from Santa Cruz Biotechnology Inc. (Santa Cruz, CA, USA); monoclonal anti-Cav-1, -integrin *β*1, and -p21 were from BD Transduction Laboratories Inc. (Lexington, KY, USA); inhibitory integrin *β*1-blocking antibody (P4C10) and anti-MT-MMP1 were from Chemicon Inc. (Temecula, CA, USA); antibodies against caspase 8, caspase 9, PARP, survivin, E2F1, phospho- and total ERK, Src and Rb were from Cell Signaling (Beverly, MA, USA). Monoclonal anti-pro and -cleaved caspase 3 were purchased from Imgenex (San Diego, CA, USA). Monoclonal anti-carcinoembryonic antigen (CEA) was from ABCAM Inc. (Cambridge, MA, USA). Specific inhibitor PP2 and U0126 were procured from Calbiochem (San Diego, CA, USA). 5-Aza-2′-deoxycytidine (5-aza-dc) was from Sigma (Missouri, MO, USA). Alexa Fluor 488 and 568 conjugated goat anti-mouse and goat anti-rabbit, respectively, were from Molecular Probes (Eugene, OR, USA). Annexin V and 7-amino-actinomycin D were purchased from BD PharMingen (San Diego, CA, USA). Detection of apoptosis was performed with terminal transferase dUTP nick end labelling (TUNEL) kit (Promega, Madison, WI, USA).

### Immunohistochemistry

Antigen retrieval was achieved by microwaving the deparaffinised formalin-fixed human tumour tissue sections. Sections were incubated with 1 : 200 dilution of anti-Cav-1 at 4°C overnight. Detection was achieved with the Vector avidin–biotin complex system (Vector Laboratories, Burlingame, CA, USA). For Cav-1 staining, rabbit IgG was used as a negative control and staining of endothelial cells was used as a positive control. The intensity of immunohistochemical staining in human tissues was measured using a numerical scale (0=no expression, 1^+^=weak expression, 2^+^=moderate expression, and 3^+^=strong expression which is similar to or stronger than the staining of endothelial cells) and quantified as Weight Index (WI=% positive staining (>0) in tumour × intensity score). Scoring was performed independently by two pathologists (SM and HJCS). An average of the two readings was used for statistical analysis.

### Stable transfection of Cav-1 cDNA in HNSCC cells

The plasmid lenti-cav-1 was constructed following the protocol described previously (Liu *et al*, 2006). Briefly, the human *cav-1* cDNA was amplified by standard reverse transcription–PCR from RNA extracted from 686LN cells using the following primers:

*Cav-1* sense: 5′-GACTAGTGCCGCCACCATGTCTGGGGGCAAATACGTAG-3′,

reverse: 5′-CGGGCCCTTATATTTCTTTCTGCAAGTTGATGCGG-3′.

The cav-1 cDNA was then ligated into the pT-easy vector (Promega) as pTeasy-cav-1 following the manufacturer's protocol. pT-easy-cav-1 was cut with *Spe*I and *Apa*I, and the released fragment containing *cav-1* cDNA was then cloned into the digested pLenti6/V5 vector and the resulting construct was named CavS. The coding region of *cav-1* cDNA was confirmed by sequencing analysis. The empty pLenti6/V5 vector was used as a control. Lentiviral production and titre determination were previously described (Liu *et al*, 2006). For infection, virus was added to M4 cells at a multiplicity of infection of 10, and the cells were then subjected to selection with 25 *μ*g ml^−1^ blasticidin 24 h post-infection. Fourteen days later, the cell clones were picked and screened for Cav-1 expression by immunoblotting.

### HNSCC metastatic xenograft mouse model

The animal experiment was approved by the Animal Care and Use Committee of Emory University. Twenty-two nude mice (athymic nu/nu, Taconic, NY, USA) aged 4–6 weeks (about 20 g body weight) were randomly divided into two groups. Each animal was injected with 1 × 10^6^ EV or CavS cells suspended in 0.05 ml of Hanks-buffered saline into the submandibular to mylohyoid muscle as described previously (Zhang *et al*, 2002). The xenograft tumours were measured three times per week. After mice were killed, cervical lymph nodes and lungs were collected, fixed immediately in 10% buffered formalin, and embedded in paraffin. Tissue sections were stained with hematoxylin–eosin and anti-CEA antibody. Lymph node and lung metastases were identified by a pathologist (SM).

### Cell proliferation sulforhodamine B (SRB) assay

Cells were seeded at 2 × 10^3^ per well in 96-well plates and incubated for 7 days with four repeats per treatment. After fixation in 10% trichloroacetic acid for 1 h at 4°C, the cells were stained with 0.4% SRB in 1% acetic acid for 10 min followed by washing with 1% acetic acid and air-drying. Sulforhodamine B bound to cellular protein was dissolved in 10 mM Tris-HCl (pH 10.5). The protein level was measured by spectrophotometry at 492 nm and expressed as number of cells proportional to protein level. Experiments were repeated four times.

### Matrigel invasion assay

Matrigel was purchased from Becton Dickinson Biosciences Discovery Labware (Bedford, MA, USA). The Matrigel invasion assay has been described previously (Zhang *et al*, 2002). Briefly, 2.5 × 10^4^ cells per well were seeded in triplicate with serum-free medium containing 0.1% BSA in the invasion chamber (BD Biosciences, Bedford, MA, USA) precoated with 27.2 *μ*g per chamber of Matrigel for 8 h before the NIH-3T3-conditioned medium was added to the lower chamber. In some experimental groups, cells were preincubated with mouse IgG, integrin *β*1 neutralising antibody (0.5 *μ*g ml^−1^), PP2, or U0126 at 5 *μ*M for 2 h at room temperature before being added to the wells. After 36–40 h of incubation, the upper membrane (8-*μ*m pore size) was gently scrubbed with a cotton swab. The invaded cells in the lower membrane were stained with the Hema-3 kit, following the manufacturer's instructions (Fisher Scientific, Pittsburgh, PA, USA). The number of invaded cells was expressed as the sum of 10 random fields under the microscope at × 200 magnification. Experiments were repeated three times.

### Demethylation assay

The parental 686LN and its metastatic derivatives from the third and fourth rounds of *in vivo* selection 686LN-M3s and 686LN-M4s, respectively, were treated with or without 5-aza-2′deoxycytidine (5-aza-dc, 5 *μ*M) for 4 days. Total protein of the lysates was analysed for Cav-1 with G3PDH as a loading control by immunoblotting. The experiment was repeated twice.

### Anoikis assay

To prevent cell adhesion, HNSCC cells were cultured onto a plastic bacteriological Petri dish precoated with poly-HEMA (Fiucci *et al*, 2002). Cells were plated at a density of 2 × 10^6^ per well in a six-well plate for 24–48 h. The survival of attached and suspended cells was determined by flow cytometry analysis of annexin V and 7-amino-actinomycin D staining. Experiments were performed in triplicate and repeated twice.

For integrin *β*1-blocking antibody experiment, serum-starved cells were preincubated with control mouse IgG or the blocking antibody (P4C10, 0.5 or 1.0 *μ*g ml^−1^) for 2 h at room temperature before they were suspended for 48 h. The suspended cells were then placed onto 0.01% of poly-lysine-coated glass slides and fixed with 4% paraformaldehyde and 0.5% Triton-X 100 for 10 min at room temperature. Double immunofluorescence staining is described in the following section. Apoptosis was assessed by performing the TUNEL assay according to the manufacturer's instructions (Promega).

### Immunoblotting, coimmunoprecipitation, and immunofluorescence analyses

Cell lysates containing equal amounts of protein were separated by 12% SDS–PAGE. Immunoblotting was carried out according to a standard procedure. Protein assays were performed using a modified Lowry procedure with a commercially available kit (Bio-Rad, Hercules, CA, USA). For coimmunoprecipitation, about 500 *μ*g of lysate was precleared with recombinant protein G-agarose (GIBCOBRL, Carlsbad, CA, USA) for 4 h at 4°C, incubated with 5 *μ*g of antibody or non-immunised rabbit or mouse IgG precomplexed with protein G-agarose overnight. Denatured samples were separated by 12% SDS–PAGE.

For immunofluorescence staining, cells grown on coverslips were fixed with warm PHEMO buffer (68 mM PIPES, 25 mM HEPES, pH6.9, 15 mM EGTA, 3 mM MgCl_2_, 10% (vol/vol) DMSO) containing 3.7% formaldehyde, 0.05% glutaraldehyde, and 0.5% Triton X-100 for 10 min and incubated with primary antibody and fluor-conjugated secondary antibodies. Images were taken with Zeiss LSM510 META confocal microscope.

### Statistical analysis

For human tissues, expression values were averaged to yield a single expression value when multiple metastatic nodes were collected on a given individual. Nonparametric methods using the Kruskal–Wallis and Mann–Whitney tests were performed to compare WI among different groups. Wilcoxon signed-rank test was applied for comparing paired samples Tu-1 and LNM-1. The incidence of metastases was analysed by *χ*^2^ analysis or Fisher exact test.

The statistical significance of treatment of cells in cell growth, invasion, anoikis assay, and the number of metastatic cells was assessed using the Student's *t*-test. *P*<0.05 was considered statistically significant in all analyses.

## Results

### Expression of Cav-1 in human HNSCC tumours

**Figure 1:**
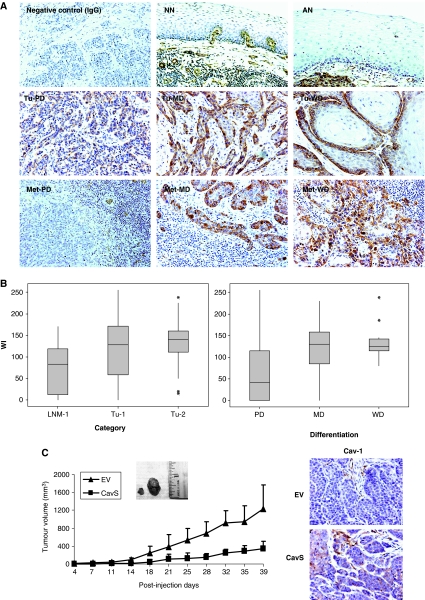
Immunohistochemical staining of Cav-1 in HNSCC specimens. (**A**) Immunohistochemical staining of Cav-1 in NN and AN (top), PD, MD, and WD of primary tumours (middle) and of lymph node metastases (bottom). Staining with IgG was used as a negative control. Magnification was × 200. (**B**) Quantified analysis of Cav-1 expression with bar graph presentation of WI of Cav-1 *vs* tumour category and differentiation in tumour samples including Tu-1, -2, and LNM-1. The line within the bar represents the median value and ^*^ represents individual data point. Expression of Cav-1 was significantly downregulated in LNM-1 compared to primary tumours (*P*<0.0001). Expression of Cav-1 was significantly lower in PD category than in MD and WD (*P*<0.0001). (**C**) Growth of tumours in animals injected with EV cells or CavS cells. Tumour volume (mm^3^) was recorded on every other 3 days post-tumour cell injection. Inserted figure shows the representative tumour size from tumours bearing CavS (left) and EV (right) cells. CavS tumour volume was substantially reduced compared with EV tumours (*P*=0.0003 by day 25, *P*=0.003 by day 39). The right panel shows representative immunohistochemistry staining of Cav-1 in EV and CavS-bearing tumours.

The downregulation of Cav-1 observed in cell lines derived from the HNSCC metastatic mouse model (Zhang *et al*, 2002, 2006) led us to examine Cav-1 expression in primary tumours and LNM of HNSCC tissue specimens by immunohistochemical analysis. The tissue samples included three categories Tu-1 (primary tumours from patients who also had LNM), LNM-1 (LNM samples from the Tu-1 patients), and Tu-2 (primary tumours from patients who did not have LNM; see Materials and Methods for details). The specificity of Cav-1 immunoreactivity was examined by using endothelium known to be abundant in Cav-1 as a positive control and rabbit IgG as a negative control (Figure 1A top). The tumour adjacent-normal (AN) tissues and 11 normal epithelial tissues from non-cancer patients (NN) were also examined as the normal cell controls (Figure 1A top). Caveolin-1 was mainly located at the membrane and in the cytoplasm of the tumour cells as well as in the endothelial cells. Expression of Cav-1 was barely detected in the normal epithelium. In tumour tissues, a typical peripheral staining pattern (Figure 1A middle and bottom) was frequently observed in which Cav-1 immunoreactivity was seen in only the outer cell layer of the nest, whereas the rest of the keratinocytes and necrotic cells were negative.

Quantitative analysis of Tu-1, LNM-1, and Tu-2 samples revealed that expression of Cav-1 was significantly downregulated in LNM-1 compared with primary tumours (median WI=82.5 for LNM-1 *vs* median WI=127 for Tu-1 and 140 for Tu-2, *P*<0.0001) (Figure 1B left). Among 30 PD, 67 MD, and 17 WD tumour samples, the expression of Cav-1 was lowest in the PD category (*P*<0.0001) (Figure 1B, right). Table 1 shows the number of cases categorised by tumour differentiation status and tumour sites. It is noticed that LNM-1 had more cases of PD than Tu-1 and Tu-2. Pairwise comparison detected significantly lower Cav-1 expression in LNM-1 compared with Tu-1 samples (*n*=31, *P*=0.0002). Analysis of patient clinical information revealed that the WI of Cav-1 inversely correlated with N-stages identified by histology (N positive=N1+N2+N3, *n*=18, and N negative=N0, *n*=22, *P*=0.043), but did not correlate with T-stage, tumour site, patient age, or survival rate.

### Effect of Cav-1 overexpression on tumorigenicity and metastasis *in vivo*

The role of Cav-1 expression in tumorigenesis and metastasis was investigated by establishing stable M4 clones infected with lentivirus expressing a full-length *cav-1* cDNA (CavS). Stable cell lines transfected with the empty vector (EV) were used as control. The level of Cav-1 protein expression in CavS cells was comparable to that in the parental 686LN cells with low metastatic potential (data not shown).

To determine the effect of restoring Cav-1 expression in metastatic cells on tumorigenesis and metastasis development, 22 nude mice were injected with 1 × 10^6^ EV cells (*n*=12) or high Cav-1 expressing CavS cells (*n*=10) (Zhang *et al*, 2002). To detect tumour progression, metastasis development, and the level of Cav-1 expression, half of the animals were killed on day 25 post-injection and the rest were killed on day 39 post-injection. Tumour volume was substantially reduced in animals injected with CavS cells compared with those injected with EV cells (*P*=0.0003 by day 25, *P*=0.003 by day 39, Figure 1C left). By day 39, tumour weights were 1.02±0.39 and 0.26±0.16 g for tumours bearing EV and CavS cells, respectively (*P*=0.001). With endothelial cells as a positive control, Cav-1 staining was detected in all tumour cells of the CavS tumour tissues but was negative in all tumour cells of the EV tumour tissues (Figure 1C right). There was no significant difference in Cav-1 expression among tumour samples collected from two time points. All animals in both groups had identifiable LNM by days 25 and 39. When taken together, 9 out of 12 mice injected with EV cells had lung metastases, whereas 3 out of 10 mice injected with CavS cells had positive lung metastases (*P*=0.03 by *χ*^2^ and *P*=0.08 by Fisher exact test when the small sample size was considered). In addition, the number of metastatic foci identified microscopically and highlighted with CEA staining was significantly lower in animals injected with CavS cells (Table 2, *P*=0.03). TUNEL assay revealed no difference in the amount of TUNEL-positive areas, including apoptotic and necrotic signals, between EV and CavS tumours (data not shown).

### Effect of Cav-1 overexpression on cell proliferation and invasion *in vitro*

**Figure 2:**
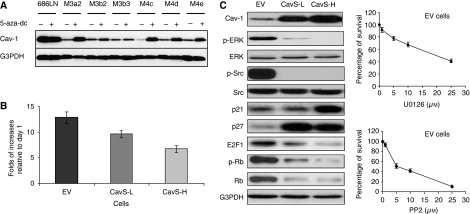
Effect of overexpression of Cav-1 on cell proliferation. (**A**) Immunoblotting analysis of Cav-1 expression from a demethylation assay. The parental 686LN and its metastatic derivatives from the third and fourth rounds of *in vivo* selection 686LN-M3s and 686LN-M4s, respectively, were treated with or without 5-aza-dc (5 *μ*M) for 4 days as described in our previous study (Zhang *et al*, 2006). The experiment was repeated twice. (**B**) Cell proliferation was expressed as fold of increases in cell number by day 7 relative to the cell number on day 1. Cell growth rate was significantly reduced in Cav-1 low expressing clones CavS-L (*P*=0.0027) and Cav-1 high expressing clones CavS-H (*P*=0.0001) compared with EV cells. The experiment was repeated three times. (**C**) Left, immunoblotting analysis of EV, CavS-L, and CavS-H. Right, SRB assay was performed by treating EV cells with various dose of U0126 or PP2 for 3 days. Percentage of survival was expressed as the number of cells relative to that in the control. Experiments were repeated four times.

Silencing of gene expression frequently occurs because of promoter hypermethylation. Thus, we investigated whether the downregulated expression of Cav-1 in the highly metastatic cell lines was due to methylation of the *cav-1* promoter or was the secondary effect of hypermethylation of certain Cav-1-regulatory genes. Treatment with the demethylation agent 5-aza-dc substantially restored the expression of Cav-1 in highly metastatic cells to a level similar to that in the parental 686LN cell (Figure 2A), but promoter hypermethylation of Cav-1 in the tested cell lines was not detected by methylation-specific PCR (Dr P Vertino, personal communication).

To understand the inhibitory effect by Cav-1 observed in the xenograft mouse model, we first examined growth rate of both EV and CavS cells. Clones of high Cav-1 expressing cells (CavS-H, similar to the levels of parental 686LN) and low Cav-1 expressing cells (CavS-L, 25–50% of the levels in CavS-H) were selected. Consistent with the observation *in vivo*, the *in vitro* cell growth rate was substantially curtailed in CavS cells compared with a pool of EV cells (Figure 2B), with 25 and 48% reductions in CavS-L (*P*=0.0027) and CavS-H (*P*=0.0001), respectively. To dissect the mechanism underlying the inhibitory actions of Cav-1 in tumorigenesis, typical Cav-1 targeting signalling molecules, including the activation of Src and ERK (Williams and Lisanti, 2005), were examined among CavS-L and CavS-H cells. Interestingly, activation of Src and ERK were substantially diminished in both low and high CavS expressing clones (Figure 2C). Some proteins involved in cell cycle regulation, such as p21, p27, E2F, and Rb, were also affected by restoration of Cav-1. To elucidate whether the ERK or Src signalling pathways play a role in growth of M4 cells, we also found that cell growth of a pool of EV cells was markedly inhibited by Src kinase inhibitor PP2, and moderately by ERK kinase inhibitor U0126 (Figure 2C).

**Figure 3:**
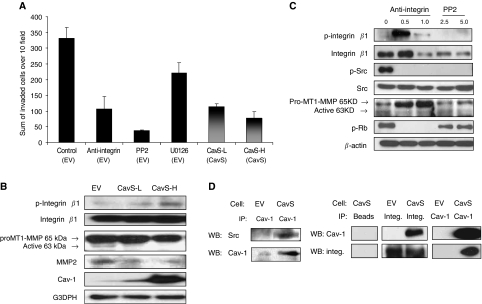
Effect of overexpression of Cav-1 on cell invasion. (**A**) Matrix gel invasion assay was performed as described in Materials and Methods. Empty vector cells were pretreated with 0.5 *μ*g ml^−1^ of IgG control or integrin *β*1-blocking antibody as well as PP2 or U0126 at 5 *μ*M for 2 h before seeding. CavS cells were also examined. Sum of invaded cells over 10 random fields was recorded. Invasion activity was significantly impaired in the treated cells and CavS cells compared with that in EV cells (*P*<0.0001). Experiments were performed three times. (**B**) Immunoblotting analysis of the proteins associated with invasion in EV, CavS-L, and CavS-H. (**C**) Immunoblotting analysis of EV cells treated with integrin *β*1-blocking antibody (*μ*g ml^−1^) or PP2 (*μ*M). (**D**) Cav-1 coimmunoprecipitated with Src and integrin *β*1. Experiments were performed three times.

We then examined the effect of restoration of Cav-1 on cell invasion by Matrigel invasion assay. Compared with a pool of EV cells, CavS-L and CavS-H cells showed 65.5 and 76.3% reduction in invasion, respectively (*P*<0.0001, Figure 3A). It was suggested that Cav-1 mediated anchorage-dependent cell growth by acting as an adaptor between integrin *β*1 and the Src family kinase Fyn (Wary *et al*, 1998). A previous study has also shown that phosphorylation of integrin *β*1 at serine 785 inhibits cell migration (Mulrooney *et al*, 2001). Thus, in addition to examining the involvement of ERK and Src signalling pathways on invasion, the overexpression of integrin *β*1 in M4 cells compared with their parental 686LN cells (Zhang *et al*, 2006) led us to investigate whether integrin *β*1-dependent functions could be modulated by Cav-1. The Matrigel invasion assay was performed with EV cells treated with integrin *β*1-blocking antibody at 0.5 *μ*g ml^−1^, PP2 and U0126 at 5 *μ*M along with CavS-L and CavS-H cells. These concentrations did not generate noticeable apoptosis (data not shown). Similar to our observations on cell proliferation, the invasion activity of EV cells was markedly suppressed by the application of PP2 and integrin *β*1-blocking antibody, and moderately inhibited by U0126 (Figure 3A).

The suppression of invasion activity in CavS cells was correlated with the expression of Cav-1 protein, and associated with increased serine 785 phosphorylation of integrin *β*1 and decreased active MT1-MMP and MMP2 (Figure 3B) in addition to the substantially suppressed activation of Src and ERK (Figure 2C). Similarly, immunoblotting analysis revealed that inhibited invasion activity of EV cells by integrin *β*1-blocking antibody was associated with markedly increased phosphorylated integrin *β*1 (ser785) at 0.5 *μ*g ml^−1^, completely diminished activation of Src, Rb and inhibited active MT1-MMP (Figure 3C). PP2 completely diminished activation of Src, moderately suppressed the active MT1-MMP at a higher dose and reduced phospho-Rb without affecting phosphorylation of integrin *β*1 (ser785). Furthermore, we indeed observed that Cav-1 could coimmunoprecipitate with integrin *β*1 and Src in CavS cells (pool of CavS-H and CavS-L clones, Figure 3D) and the parental 686LN cells (data not shown).

### Overexpression of Cav-1 on anoikis

**Figure 4:**
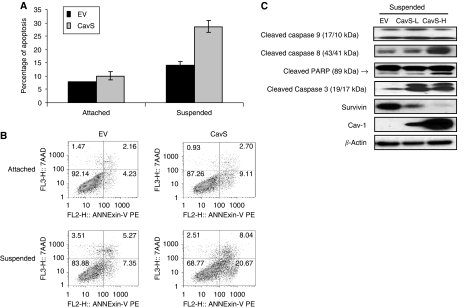
Effect of reintroducing Cav-1 on anoikis. (**A**) Flow cytometry analysis of apoptosis between attached and suspended EV and CavS cells (various high and low Cav-1 expressing clones). Cells were allowed to attach or suspend in the culture dishes for 48 h. The survival of attached and suspended cells was determined by flow cytometric analysis of annexin V and 7-amino-actinomycin D staining. Attachment or suspension had significant different effects on apoptosis of both cells (*P*<0.0001). CavS cells exhibited 23.0% (*P*=0.19) and 51.4% (*P*<0.01) greater apoptosis under attachment and suspension conditions, respectively, than the EV cells. (**B**) Representative of flow cytometry analysis shows distribution of apoptotic cells in the attached and suspended conditions. (**C**) Activation of caspases detected by immunoblotting in EV, CavS-L, and CavS-H cells under 48 h of suspended or attached condition. Figure was shown as representative from three independent experiments.

M4 cells have acquired anoikis-resistance compared with parental 686LN cells (Zhang *et al*, 2006). When M4 cells were in suspension, they tended to form aggregates to sustain survival. Their lack of E-cadherin and Cav-1 while possessing high levels of integrin *β*1(Zhang *et al*, 2006) led us to hypothesise that loss of Cav-1 may facilitate integrin *β*1-mediated cell–cell contact by forming aggregates, leading to escape from anoikis. To test this hypothesis, we first performed anoikis assay to compare EV and CavS cells. When EV and CavS cells were deprived from attachment to matrix for 48 h, CavS cells exhibited 51.4% (*P*<0.01) greater apoptosis than EV cells. Figure 4B shows a representative distribution of the apoptotic cell population from the flow cytometry analysis. As the result of marked increase in anoikis, higher levels of cleaved caspases 3, 8, and PARP were detected in CavS-H cells than in EV cells (Figure 4C). Survivin protein expression was barely detectable in CavS-H cells under suspension conditions (Figure 4C).

**Figure 5:**
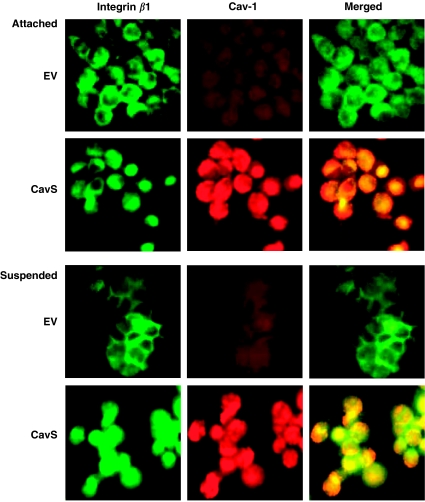
The effect of direct association of Cav-1 with integrin *β*1 on integrin *β*1-mediated survival. Immunofluorescence staining of integrin *β*1 (the first column), Cav-1 (middle column), and the merged images (the right column) of EV and CavS cells under attached (top) or suspended condition (bottom). Image was taken with confocal microscopy (magnification × 630).

**Figure 6:**
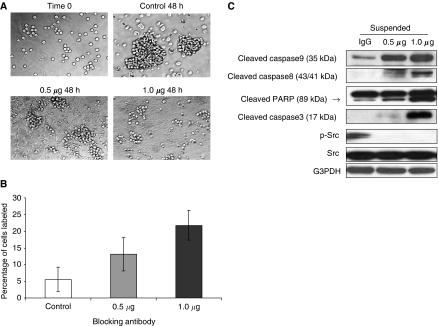
Integrin *β*1-mediated invasion and survival in cell suspension condition. (**A**) Bright field images of EV cells treated with mouse IgG control, functional-blocking antibody of integrin *β*1 at 0.5 *μ*g ml^−1^ or 1.0 *μ*g ml^−1^ for 48 h (magnification × 40). (**B**) TUNEL-positive cells were quantified and expressed as percentage relative to the number of total cells labelled by DAPI. Significant increase of TUNEL-labelled nuclei were observed in all treated cells (*P*=0.04). No difference was detected between two doses. (**C**) Immunoblotting analysis of EV cells treated with integrin *β*1-blocking antibody at 0.5 *μ*g ml^−1^ or 1.0 *μ*g ml^−1^ for 48 h. Experiments were performed three times.

We then asked whether the direct interaction of Cav-1 with integrin *β*1 may modulate integrin *β*1-mediated survival in M4 cells. We observed that the formation of aggregates in suspended CavS cells was less than that in EV cells (data not shown). Immunofluorescence staining revealed that a membranous distribution of integrin *β*1 was detected in the attached cells, but intensely accumulated at cell–cell junctions in the suspended EV cells (Figure 5). In the presence of CavS cells, integrin *β*1 exhibited noticeable internalisation and colocalised with Cav-1 protein both in the membrane and the cytoplasm under either attached or suspension conditions. Similarly, when integrin *β*1-blocking antibody was applied to EV cells in suspension for 48 h, it was found that aggregate formation was substantially inhibited (Figure 6A). Integrin *β*1-blocking antibody induced significant dose-dependent increases in apoptosis detected by TUNEL assay (Figure 6B, *P*=0.04), increased cleavage of caspases 9, 8, 3 and PARP, and decreased phosphorylated Src compared with cells treated with IgG control (Figure 6C).

## Discussion

**Figure 7:**
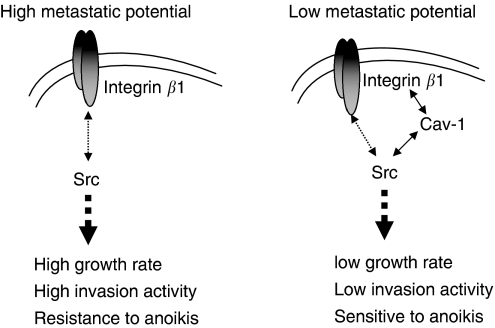
Schematic model of Cav-1 in inhibiting tumour growth and metastatic progression. In highly metastatic cells, loss of Cav-1 may facilitate activation of integrin *β*1/Src-mediated biological activities, including cell growth, invasion, and anoikis resistance. Restoration of Cav-1 could reverse the aggressive behaviour of highly metastatic tumour cells by modulating the vital integrin *β*1/Src-mediated cell–cell and cell–matrix machineries, leading to suppression of tumour growth and metastasis potential. Solid line and dashed line represent direct and indirect effect, respectively.

Studies supporting Cav-1 as a negative regulator of tumour progression have been thoroughly reviewed (Williams and Lisanti, 2005). However, concrete evidence is lacking for a definite role of Cav-1 not only in acquiring metastasis but also in metastatic progression. Thus, this study was designed to examine the effect of Cav-1 overexpression on tumour progression and metastasis potential of HNSCC. Examination of human HNSCC tumour tissues revealed significantly lower levels of Cav-1 in LNM than in the primary tumours with and without LNM. This indicates that the presence of Cav-1 in cells with low metastatic potential and the loss of Cav-1 in highly metastatic cells observed in our model may represent HNSCC metastasis development. Thus by using this highly metastasis xenograft mouse model, we were able to show that restoration of Cav-1 protein expression substantially reduced tumour growth and inhibited lung metastasis. Further *in vitro* studies revealed that restoration of Cav-1 protein significantly inhibited metastatic cell growth and invasion, and sensitised metastatic cells to anoikis, possibly through the interactions of Cav-1 with integrin *β*1 and Src. On the basis of these findings, it is plausible to suggest that restoration of Cav-1 could reverse the aggressive behaviour of highly metastatic tumour cells by modulating the vital integrin *β*1/Src-mediated cell–cell and cell–matrix machineries, leading to suppression of tumour growth and metastasis potential in late stage of tumour development (Figure 7).

Loss of Cav-1 expression has been reported in oral SCC metastatic lesions (Hung *et al*, 2003). The absence of significant hypermethylation in *cav-1* promoter and the restoration of Cav-1 expression after 4 days of demethylation treatment (Figure 2A) indicate that the loss of Cav-1 expression could be a secondary effect of hypermethylation of certain Cav-1 regulatory genes. We did not observe any significant correlation of Cav-1 expression with tumour stage and survival rate, as we were unable to stratify the patients due to treatment and limited sample size. However, we were able to assess the dynamic changes in Cav-1 protein expression during metastasis by performing pairwise comparisons of Cav-1 expression levels between primary tumours and LNM from the same patient. The analysis revealed significant downregulation of Cav-1 in LNM and that Cav-1 expression was inversely associated with N-stage and positively with tumour differentiation. In contrast to our observation, a study of oesophageal SCCs found that overexpression of Cav-1 was associated with LNM and a worse prognosis after surgery (Kato *et al*, 2002), suggesting that the effect of Cav-1 on metastasis progression could be tissue-specific as illustrated in the contradictory cases between breast cancer (Sloan *et al*, 2004) and prostate cancer (Williams *et al*, 2005).

The interplay of Cav-1 with integrins could modulate integrin-dependent tumour cell growth and invasion. Consistent with the finding by Wary *et al* (1998), we observed that Cav-1 may be capable of mediating integrin/Src-dependent cell growth and invasion by interacting with integrin *β*1 and Src. Reduced cell growth and invasion activity in CavS cells were associated with increased phosphorylated integrin *β*1 (ser785) and completely diminished activation of Src, similar to the effect of integrin *β*1-blocking antibody and Src inhibitor PP2. Further evidence of the interaction came from the findings that Cav-1 coimmunoprecipitates with integrin *β*1 and Src, and the reduced levels of membranous integrin *β*1 probably as a result of internalisation by Cav-1 (Figure 5). This observation is consistent with the finding that increased phosphorylated integrin *β*1 (ser785) leads to reduced cell migration as a result of failure to form a focal adhesion complex with integrin *β*1 (Mulrooney *et al*, 2001). These data indicate that Cav-1 may directly affect integrin *β*1-dependent cell growth, invasion, and survival through inside-out signalling. Inhibition of EV cell growth and invasion induced by PP2 resembled the effect of integrin *β*1-blocking antibody, evidenced by complete suppression of activated Src without the induction of phosphorylated integrin *β*1 (ser785), indicating that Src activation may be a downstream signalling consequence of integrin activation. Thus, the diminished phosphorylated Src in CavS cells could be a result of both the direct interference by Cav-1 and indirect consequence of the interaction between integrin *β*1 and Cav-1 (Figure 7). The pivotal roles of Src in cell growth and metastasis has been well established (Yeatman, 2004). Significant enhancement of p21 and p27 levels associated with reduced cell growth in CavS cells compared with EV cells could be further supported by a recent study that Src stabilises p27 protein (Chu *et al*, 2007).

This study has demonstrated that Cav-1 suppresses anoikis resistance, possibly by interfering integrin-mediated cell–cell contact-dependent survival mechanism (Yu *et al*, 2000; Grossmann, 2002). It has been reported that human oral SCC cells could evade p53-mediated anoikis by forming aggregates through *α*v integrin-fibronectin binding (Zhang *et al*, 2004b). Upregulated integrin *β*1 in the metastatic HNSCC cells is associated with increased anoikis resistance, invasion activity and much stronger adhesion to fibronectin, a ligand for integrin *β*1, compared with their parental 686LN cells (Zhang *et al*, 2002, 2006). The application of integrin *β*1 functional-blocking antibody effectively abolished cell aggregates, inducing substantial anoikis associated with completely suppressed activated Src, suggesting that anoikis resistance in M4 cells was achieved by an integrin *β*1/Src-mediated cell–cell contact survival mechanism (Figure 6). The interaction of Cav-1 with integrin *β*1 and with Src may reduce the cell–cell contact (Figure 5), thus diminishing the survival capability in CavS cells under suspension condition, as greater apoptosis was only observed in CavS cells upon suspension but not under attachment conditions (Figure 4A–C). Furthermore, the downregulation of survivin in suspended CavS cells could also account for their reduced anoikis resistance (Figure 4). The difference in the pattern of cleaved caspases 8 and 9 between the EV cells treated with integrin *β*1-blocking antibody (Figure 6C) and suspended CavS cells (Figure 4C) suggests that the interaction of Cav-1 with integrin *β*1 may not be sufficient to activate the intrinsic apoptosis pathway. This inside-out signalling could be different from the outside-in signalling induced by integrin *β*1-blocking antibody. The signalling pathways activated under suspension conditions are different from those under attachment conditions, as there are no noticeable changes in phosphorylated integrin *β*1 (ser785) in suspended CavS and EV cells treated with or without integrin *β*1-blocking antibody (data not shown). There is a discrepancy between our observations with the findings that MCF-7 cells transfected with *cav-1* cDNA exhibited increased survival after detachment (Ravid *et al*, 2005). This could be that MCF-7 cells do not have metastatic potential. Thus, the significantly lower incidence of lung metastases in the CavS tumour-bearing mice than in the EV-bearing mice could be the result of greater anoikis in CavS cells occurring in the blood circulation before their landing in the lung. Taken together, our findings are consistent with the current views that upregulated expression of integrin *β*1 may play a pivotal role in metastasis development (Janes and Watt, 2006). The mechanisms by which expression of Cav-1 and its interaction with integrins affects cell survival pathways remain to be studied.

By using a late stage metastasis model, this study has revealed a novel finding that Cav-1 has differential effect on lymph node and lung metastasis. Caveolin-1 could curtail distance metastasis, consistent with our *in vitro* observation that Cav-1 sensitises metastatic cells to anoikis but not under attached condition. The lack of difference in the incidence of LNM between EV and CavS expressing xenograft mice suggests that M4 cells may have adapted to be destined to metastasise to the lymph node in mice, overcoming the inhibition exerted by Cav-1. Although regional metastasis poses as an unfavourable prognostic factor, it is the distant metastasis that is always incurable. Thus, the differential effect of Cav-1 observed in the study may help shed lights on its conflicting views of prognostic indicator. There were more than 5000 genes significantly altered in the highly metastatic M4 cells compared with the parental low-metastasis 686LN cells (Zhang *et al*, 2006), including the loss of E-cadherin and p53, the known key players in tumour suppression and metastasis. However, simply restoring Cav-1 expression could indeed suppress tumour growth and reduce distant metastasis *in vivo*. In conclusion, our data suggested that Cav-1 could play an inhibitory role in tumorigenesis and lung metastasis of HNSCC through regulating integrin *β*1- and Src-mediated cell–cell and cell–matrix interactions.

## Figures and Tables

**Table 1:** Number of cases in tumour differentiation status and tumor sites

**Total=114**	**WD=17**	**MD=67**	**PD=30**
T1=34	3	20	11
T2=40	10	27	3
LNM-1=40	4	20	16

**Table 2:** Number of invaded tumour cells identified in the lung tissues

**Days post-injection**	**EV animal tag no.**	**Number of cells**	**CavS animal tag no.**	**Number of cells**
25	159	0	166	0
	174	0	169	0
	153	18	172	0
	154	13	163	1
	156	42	173	5
	158	12		
39	151	0	89	0
	91	21	161	0
	152	7	165	0
	155	61	90	2
	157	10	170	25
	160	36		

The number of metastatic foci highlighted with CEA staining was significantly lower in animals injected with CavS cells (*P*=0.03).
